# Incidence of Ewing's Sarcoma in Different Age Groups, Their Associated Features, and Its Correlation With Primary Care Interval

**DOI:** 10.7759/cureus.13986

**Published:** 2021-03-18

**Authors:** Salahuddin Khan, Zain Abid, Ghulam Haider, Neelma Bukhari, Desaar Zehra, Madiha Hashmi, Masooma Abid, Umer Ibrahim

**Affiliations:** 1 Oncology, Jinnah Postgraduate Medical Center, Karachi, PAK; 2 Emergency Department, National Medical Center, Karachi, PAK

**Keywords:** age pattern, site, lymphadenopathy, metastasis, painful swelling, primary care physician, prognosis, differential

## Abstract

Introduction

Primary care interval is the time duration from a patient’s first presentation to the final diagnosis. Ewing’s sarcoma is a rare small round blue cell bone tumor originating from neuroectoderm and undifferentiated neuroepithelial cells, having an annual incidence of approximately one case per million in the United States. In this study, we analyzed the age pattern among patients diagnosed with Ewing’s sarcoma undergoing management, along with associated features including involved site, regional lymphadenopathy, and distant metastasis at the time of presentation and their correlation with the primary care interval.

Methods

This is a cross-sectional study carried out at the Oncology department of a Tertiary Care Government Hospital in Karachi, Pakistan. The duration of our study was from January 2020 to December 2020. During this period, all patients with proven diagnosis of Ewing’s sarcoma between ages 10 years and 65 years were included in the study. All the participants of the study were divided into groups, based on the age and site of the tumor.

Results

A total of 895 cases of bone cancer were reported. Among these, 147 cases (16.4%) had Ewing’s sarcoma. Of these patients, 88 were male (60%) while 59 (40%) were female. The mean age of patients was 18.9 ± 3.2 years. Ewing’s sarcoma most commonly occurred during 15 to 20 years of age. The most common region involved was lower limb (n=76, 52%) followed by upper limb (n=63, 43%) followed by pelvis (n=8, 5.4%).

Conclusion

The peak time for the occurrence of Ewing’s sarcoma is from 15 years to 20 years of age. Regional painful swelling is the most common presenting feature in our study population. Factors causing a prolonged primary care interval include early age of onset, non-specific clinical presentation, and insufficient knowledge of the primary care physician, which results in poor prognosis. Hence, it is important to consider Ewing’s sarcoma as a differential on the first presentation especially in the high-risk age group.

## Introduction

Ewing’s sarcoma is a rare small round cell bone tumor originating from neuroectoderm and undifferentiated neuroepithelial cells, having an annual incidence of approximately one case per million in the United States. It is significantly common among adolescence and is rare among adults. Around 80% of cases are found in patients under 18 years of age and less than 1% of cases are found in adults greater than 40 years of age [1]. The known cause for Ewing’s sarcoma is chromosomal translocation at 11:22(q24:q12) which produces a combination segment of the 3’ segment of ETS FLI-1 gene and 5’ of EWS gene. The resulting protein EWS-FLI-1 activates transcription factor that favors the development of Ewing’s sarcomas [2]. Sarcomas account for 15% of bone carcinomas [3]. Sarcomas are aggressive malignancies in both children and adults. High-grade sarcomas are less responsive to radiation and chemotherapy and have a higher potential to metastasize. However, with the advancement of diagnostic modalities, patients are diagnosed at an earlier stage; therefore, long-term survival has improved [4].

Primary care interval is the time duration from the patient’s first presentation to the final diagnosis. Ewing’s sarcoma usually has a prolonged Primary Care Interval due to non-specific presenting complaints. In the younger population, it is usually confused for traumatic injury, tendinitis, or septic arthritis with many patients on long term antibiotic courses without any relief while in older patients, it is misdiagnosed as the early presentation of rheumatoid arthritis, and many patients are subjected to oral and even intra-articular steroid administration before reaching the diagnosis of Ewing’s sarcoma. Till the final diagnosis is reached, the disease has advanced and is associated with a poor prognosis and survival.

Ewing’s sarcoma can spread to other areas, making treatment and recovery more difficult. Lung, bone, and or bone marrow are the most common site of metastasis, whereas central nervous system (CNS) and lymph node metastasis is less common.

The incidence of Ewing’s sarcoma is significantly higher in Caucasians as compared to Asians and Africans. Some researchers have attributed this due to the sparse data available on the incidence of bone malignancies in Asians and Africans [5]. The initial presenting symptoms for Ewing’s sarcoma vary among individuals including lump and/or bone ache [6]. Early diagnosis increases the chances of survival and limb salvage. It is more common in children and teenagers but can occur at any age. The mean age for diagnosis is 14-15 years while increasing age at diagnosis leads to worse outcomes. Ewing’s sarcoma most often begins in the extremities followed by the pelvis, but it can occur in any bone. Less often, it can occur in the soft tissues of the chest, abdomen, and other sites.

The objective of this study is to analyze the age distribution along with major clinical features at the time of presentation including the site of involvement, distant metastasis, and regional lymphadenopathy. Identifying the high-risk age group in the Pakistani population can be a useful clue towards Ewing's sarcoma diagnosis since the clinical presentation is usually unhelpful.

We also aim to identify obstacles faced by patients in reaching the final diagnosis which includes incomplete knowledge of primary care physicians, the younger age group with the non-specific presentation, and misdiagnosis.

## Materials and methods

This is a cross-sectional study that was carried out in the Oncology department of the government’s largest tertiary care setup in Pakistan. The time period of this study was from January 2020 to December 2020, during which all patients with proven diagnosis of Ewing's sarcoma between ages 10-65 years were included. Variables included are age at diagnosis, gender, initial symptoms, method of diagnosis, histopathologic findings, site of the tumor, and stage at diagnosis. Patients have been divided into age-based groups and primary tumor sites accordingly. Informed consent was taken from the patients before including them in the study. Trained professionals were asked to fill a detailed proforma. The diagnosis was confirmed by histopathology and chromosomal translocation at 11:22(q24:q12). Unpaired t-test for continuous data and odds ratio with 95% confidence interval for categorical variables were calculated using Statistical Package for Social Sciences (SPSS) version 20 (IBM SPSS Statistics, Armonk, NY).

## Results

**Figure 1:**
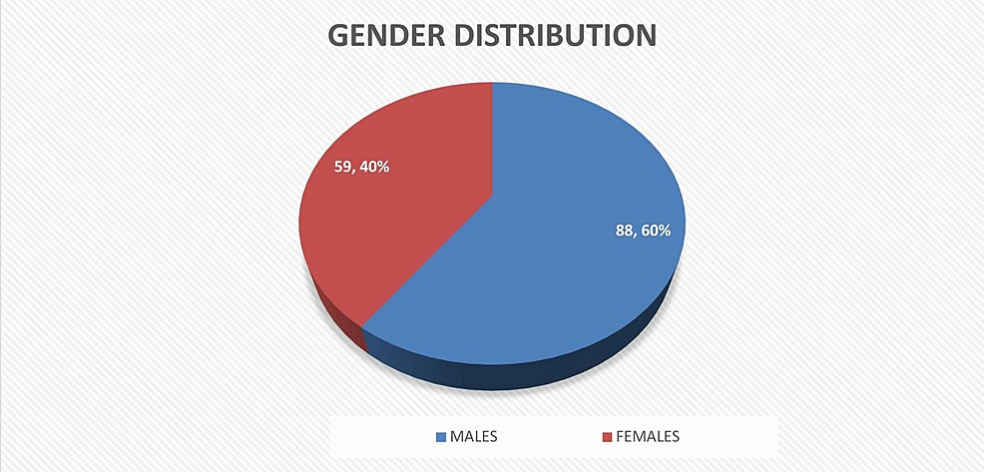
Gender distribution

During the study duration, 895 cases of bone cancer were reported out of which 147 cases (16.4%) were of Ewing’s sarcoma. Among these patients, 88 were males (60%) while 59 (40%) were females (Figure 1).

**Figure 2:**
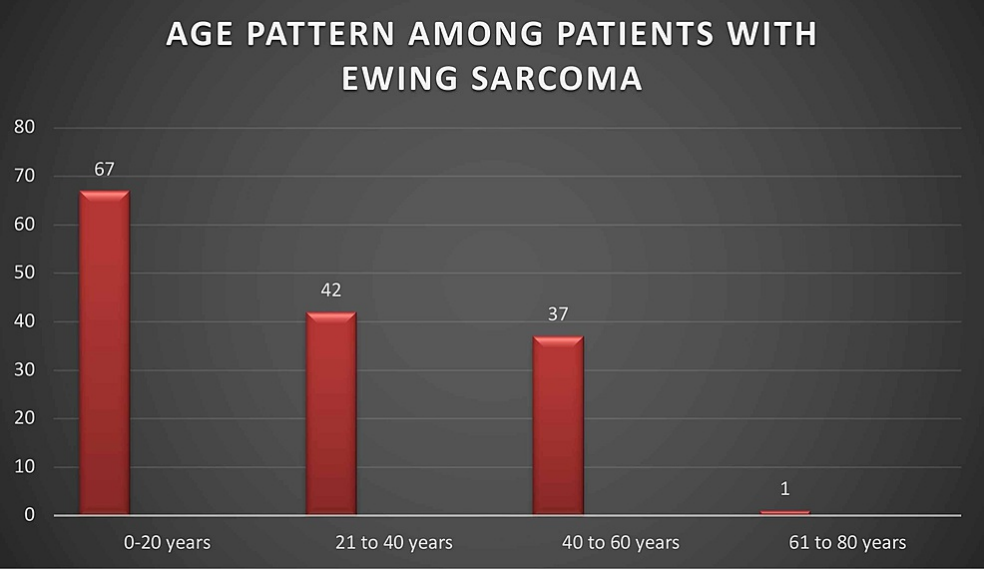
Age pattern among patients with Ewing's sarcoma

The number of cases between zero years and 20 years was 67 (45.5%), between ages 20 years and 40 years was 42 (28.5%), between ages 40 years to 60 years was 37 (25.1%), and only one patient (0.6%) was more than 60 years, whereas no patient in our study population had aged more than 65 years. Thus, Ewing’s sarcoma was found to be most common during the age of 15-20 years (Figure 2).

The most common region involved was the lower limb (n=76, 52%) with the femur as the most common site followed by the upper limb (n=63, 43%), whereas eight patients had Ewing’s sarcoma of the pelvic region (n=27, 5.4%).

In our study population, the most common presenting complaint was painful swelling. At the time of diagnosis, 10% (n=14) had distant metastasis while 23% (n=34) had regional lymph node involvement (Table 1).

**Table 1:** Demographic variables

Demographic Variables	
Total number of patients with bone tumors	n=895
Number of patients with Ewing's sarcoma	n=147
Gender distribution	
Males	n=88
Females	n=59
Age distribution	
0-20 years	n=67
21-40 years	n=42
41-60 years	n=37
61-80 years	n=1
Site involvement	
Lower limbs	n=76
Upper limbs	n=63
Pelvis	n=8
Regional lymph node involvement at presentation	n = 34
Distant metastasis at presentation	n = 14

On radiographic examination (plain radiograph and CT scan), the most common findings include the permeative process of bone/moth-eaten appearance in bone (65%) and laminated “onion skin” periosteal reaction (33%). Two percent of patients had a “sunburst” pattern on the radiographic examination which is more commonly seen in osteosarcoma.

## Discussion

Our study shows that the peak time for the occurrence of Ewing’s sarcoma is before the age of 20 years. The mean age of our study patients was 18.9 ± 3.2 years. It is more common in males and significantly higher in the extremities compared to other bones of the body. Chakraborty et al. in their study also show increased incidence among individuals aged 10-14 years of age [3]. Studies from Pakistan, Saudi Arabia, and India showed that 50% of the cases were found in the age group 11-20 years with only 12% of the cases in >30 years age group [7]. Skeletal Ewing’s sarcoma subtype is more common than soft tissue Ewing’s sarcoma, the skeletal one comprises 96% of cases worldwide. As compared to a study done in California, Asian countries had a higher ratio of Skeletal Ewing’s sarcoma [8]. A retrospective study of 101 pediatric patients suggested that localized disease had good treatment outcomes, however, side-effects can occur due to treatment. Hence long-term follow-up is recommended to check for late effects of the treatment [9].

Pediatric malignancies are usually difficult to diagnose owing to the patient’s inability to effectively describe the symptoms. Early age can be considered as a huge obstacle for diagnosing Ewing’s sarcoma which contributes to longer primary care interval, as children are usually unable to explain their symptoms which are generally attributed to trauma or infection respectively. Thus, it is imperative that when a child belonging to the age group of 12-20 years presents to a General Physician with non-specific symptoms of pain or regional swelling, Ewing’s sarcoma must be considered.

Esiashvili et al. analyzed the age-standardized incidence rate (ASIR) to compare the age-specific incidence with reference to the standard population [10]. The Surveillance, Epidemiology, and End Results (SEER) public access database includes data from various cancer care organizations in the United States. It was concluded that the ASIR per million population was highest during the years 1973-2004. Ewing’s sarcoma was reported to be 2.93 per million among individuals aged one to 19 years of age [10]. The incidence of Ewing’s sarcoma differs among populations with a higher incidence in Indians as compared to Chinese and Malay populations for both genders [3]. It has also been concluded that the age of presentation is lower in females than males [7]. Some researchers have also explained that the higher incidence of Ewing’s sarcoma in adolescence is due to the release of growth factors and sex steroids. These factors promote the growth of malignant cells. Few prenatal factors are also said to be associated with the development of Ewing’s sarcoma. These include rib anomalies, low birth weight, and peri-conceptional agricultural occupation [11]. Data from the European Ewing tumor Working Initiative of National Groups‐99‐Protocol (EURO‐E.W.I.N.G.‐99) was analyzed, and it showed that the site of the tumor and its metastasis were linked to the age of the patient. Young children aged zero to nine years with Ewing’s sarcoma had less metastatic disease at presentation and adults aged more than 24 years showed an increased propensity for tumors in the axial skeleton and pelvic region [12].

According to our study, most of Ewing’s sarcoma patients came with the presenting complaints of painful swelling (n=78, 53%) followed by pain only (n=62, 42%). This is important since these are usually non-specific symptoms leading to misdiagnosis. Another important aspect to note is that these symptoms may be accompanied by misguided trauma history with the intermittent and non-continuous presentation, thus it may further confuse the primary doctors. This results in an increased primary care interval leading to late initiation of treatment and consequently poor prognosis. Widhe and Widhe describe this as “doctor’s delay” noting that it is greater for Ewing’s sarcoma patients than osteosarcoma patients in his study [13]. Thus, primary care physicians need to be aware of Ewing’s sarcoma as a highly possible diagnosis in younger patients presenting with pain or painful swelling particularly in extremities as described in our study. Insufficient knowledge of primary care physicians can divert the management to a futile misdiagnosis leading to wastage of precious time for patients and oncologists. Hence, an oncology referral must be in the mind of the general physician when dealing with such sort of cases.

The risk of recurrent disease among patients with Ewing’s sarcoma is considered to be high. There is a 30-40% chance of relapse among patients with the primary disease [14]. Stahl et al. in their study concluded that the five-year overall survival among these patients is very poor. Some prognostic factors associated with favorable outcomes include onset after the age of two years and localized relapse [15].

## Conclusions

The primary care interval is long in Ewing's sarcoma patients. Factors causing prolonged primary care interval include early age of onset, non-specific clinical presentation, and insufficient knowledge of the primary care physician, which results in poor prognosis. Hence, it is important to spread awareness among primary care physicians regarding the common age group and clinical presentation of Ewing’s sarcoma so that they may consider it as a differential on the first presentation especially in the high-risk age group.
